# Prevalence of Dental Caries in Patients on Renal Replacement Therapy—A Systematic Review

**DOI:** 10.3390/jcm12041507

**Published:** 2023-02-14

**Authors:** Deborah Kreher, Bero Luke Vincent Ernst, Dirk Ziebolz, Rainer Haak, Jonathan de Fallois, Thomas Ebert, Gerhard Schmalz

**Affiliations:** 1 Department of Cariology, Endodontology and Periodontology, University of Leipzig, 04109 Leipzig, Germany; 2 Medical Department III—Endocrinology, Nephrology, Rheumatology, University of Leipzig, 04109 Leipzig, Germany

**Keywords:** oral health, dental caries, renal replacement therapy, hemodialysis, kidney transplantation

## Abstract

Patients under renal replacement therapy (RRT) often show oral problems, including dry mouth, periodontal and dental diseases. This systematic review aimed to evaluate the caries burden in patients on RRT. Therefore, a systematic literature search based on the databases PubMed, Web of Science and Scopus was performed by two independent individuals in August 2022. Search terms were: “caries” AND “dialysis”, “caries” AND “renal replacement therapy”, “caries” AND “kidney”. The systematic process was complemented by manual search. Studies on adult patients (age ≥ 18 years), treated by any form of RRT and explicitly reporting caries prevalence or incidence were checked for their eligibility and subsequently analyzed qualitatively. For all included studies, a quality appraisal was applied. From the systematic search, 653 studies were identified, of which 33 clinical investigations were included in the qualitative analysis. The majority (31 studies) of all included patients underwent hemodialysis (HD), with a sample size between 28 and 512 participants. Eleven studies investigated a healthy control group. Oral examinations were heterogeneous across studies; the caries burden was primarily assessed by decayed-(D-T), missing- and filled-teeth index (DMF-T). The number of decayed teeth ranged between 0.7 and 3.87 across studies. Only six out of these 11 studies found significant differences in caries prevalence/incidence between RRT and controls, whereby only four studies confirmed worse caries burden in RRT individuals. No information was provided on caries stadium (initial caries, advanced caries, invasive treatment need), caries activity or location (e.g., root caries) across studies. Most of the included studies were assessed to be of moderate quality. In conclusion, patients on RRT suffer from a high prevalence of dental caries. Alongside a need for further research in the field, improved, multidisciplinary, patient-centered dental care concepts are required to support dental and overall oral health in individuals on RRT.

## 1. Introduction

Dental caries is one of the most prevalent chronic diseases worldwide, and is among the most common oral diseases [1,2,3]. Caries is a dynamic process of biofilm-related, substrate-driven (carbohydrates) demineralization and subsequent decay of hard tooth tissues, including enamel and dentine [4]. Depending on exposition, dental caries, which develops primarily in elderly individuals, can affect the crown of the tooth, as well as the root surfaces; moreover, the progression of carious lesions can lead to infections in the dental pulp, apical bone and, in consequence, tooth-surrounding tissues [4].

Several risk factors for dental caries have been revealed, including carbohydrate intake, oral hygiene and local, tooth-related factors [4]. On the other hand, several systemic diseases, and their manifestations in the oral cavity, can be associated with caries development and progression [5]. Thereby, the composition as well as the quantity of saliva, which can be related to different systemic diseases, appear especially relevant [5]. In this context, xerostomia, which influences the occurrence of dental caries, is an important factor [6]. Accordingly, it appears unsurprising that dental caries is an important topic for patients with chronic kidney disease (CKD), especially those on renal replacement therapy (RRT), including hemodialysis (HD), peritoneal dialysis (PD) and kidney transplantation (KTx) [7]. Consequently, the recent literature has addressed this topic under different viewpoints: patients under HD were found to suffer from a high prevalence of dental caries, which worsened with increasing vintage time [8]. Furthermore, different microbiological issues regarding *Lactobacillus* and *Streptococcus mutans*, which are relevant cariogenic bacteria, have been investigated, especially under consideration of co-morbidities such as diabetes mellitus [9]. Moreover, a three-year cohort study of patients with HD showed that the all-cause mortality of the included individuals was related to their caries prevalence [10]. The latter underlines the potential relevance of oral health, especially the caries burden in individuals on RRT. One further issue, which supports the scientific interest on dental caries of patients on RRT, is the patient’s perspective, i.e., the oral health-related quality of life; thereby, oral conditions, alongside RRT-related general parameters, can potentially affect the general and oral health-related quality of life in patients on RRT [11].

Although the body of literature in this research area is growing, several questions remain unanswered, including highly clinically relevant issues such as root caries. Additionally, the variety of studies performed might result in a certain heterogeneity of results. Therefore, this systematic review aimed to evaluate the prevalence of dental caries in adult patients undergoing RRT and its comparison with healthy subjects, if available. Thereby, the characteristics and results of the available studies, their quality and a potential comparison of caries prevalence between RRT and healthy control individuals should be assessed. It has been hypothesized that patients on RRT had a high prevalence of dental caries, which is assumed to be higher than in healthy individuals.

## 2. Methods

The authors followed criteria established in the Preferred Reporting Items for Systematic Reviews and Meta-Analyses (PRISMA) guidelines for this review [12].

### 2.1. Focused Question

In this systematic review, the PICO (patients, intervention, comparison and outcome) question was whether patients undergoing RRT would show a higher caries prevalence than healthy individuals. Therefore, patients were individuals undergoing any form of RRT, including haemodialysis and peritoneal dialysis as well as transplantation. An intervention was not assessed. For comparison, either a healthy control/comparison group or available references should be used. As outcome, any form of caries assessment was defined. It was hypothesized that patients on RRT would show a higher caries prevalence than healthy individuals.

### 2.2. Eligibility Criteria

Studies which included adult patients (minimum age 18 years) requiring RRT were included in the current systematic review. It was mandatory that the occurrence of caries was explicitly described and that a full-text in English was available. Furthermore, any type of clinical study (cross-sectional, retrospective, prospective, randomized trial) was considered. Both studies with and without any form of control or comparison group (e.g., healthy controls, kidney failure without dialysis) were included in this systematic review.

Exclusion criteria were in vitro and animal designs and studies examining children and/or adolescents (age below 18) as well as an absence of explicit reporting on dental caries in the cohort.

### 2.3. Search Strategy

The literature search, based on the databases of PubMed, Web of Science and Scopus, was performed by two independent individuals in August 2022 (date of literature search: 15th August 2022). The used search terms were: “caries” AND “dialysis”, “caries” AND “renal replacement therapy”, “caries” AND “kidney”. The systematic process was complemented by manual search. Subsequently, the studies were checked for their suitability. Studies published until 15th August were considered, without defining a lower limit regarding the year of publication.

### 2.4. Data Extraction

The following information was extracted from the included studies:

- year of publication, study type, country
- number of participants, sex, age, type of RRT, duration of treatment
- caries, tooth loss/remaining teeth/dentures, oral hygiene parameters, glomerular filtration rate, laboratory parameters, saliva parameters, bacteria/-metabolism
- presence of a control group, sex, age

The systematic search as well as study selection and qualitative analysis was performed by two independent reviewers.

### 2.5. Quality Assessment

The 11-item checklist for cross-sectional studies recommended by the Agency for Healthcare Research and Quality (AHRQ) was used to assess the quality of the studies included [13]. To determine a total score for evaluation, “No” or “Unclear” responses were assigned 0 points per question, while “Yes” responses were assigned one point. For each study, a total score of 0–3 indicated low quality, a score of 4–7 indicated medium quality, and a score of 8–11 indicated high quality. Quality scores were independently assessed by the first authors (DK, BE) and the senior author (GS). Disagreements were discussed and resolved among the authors.

## 3. Results

### 3.1. Search Findings

**Figure 1:**
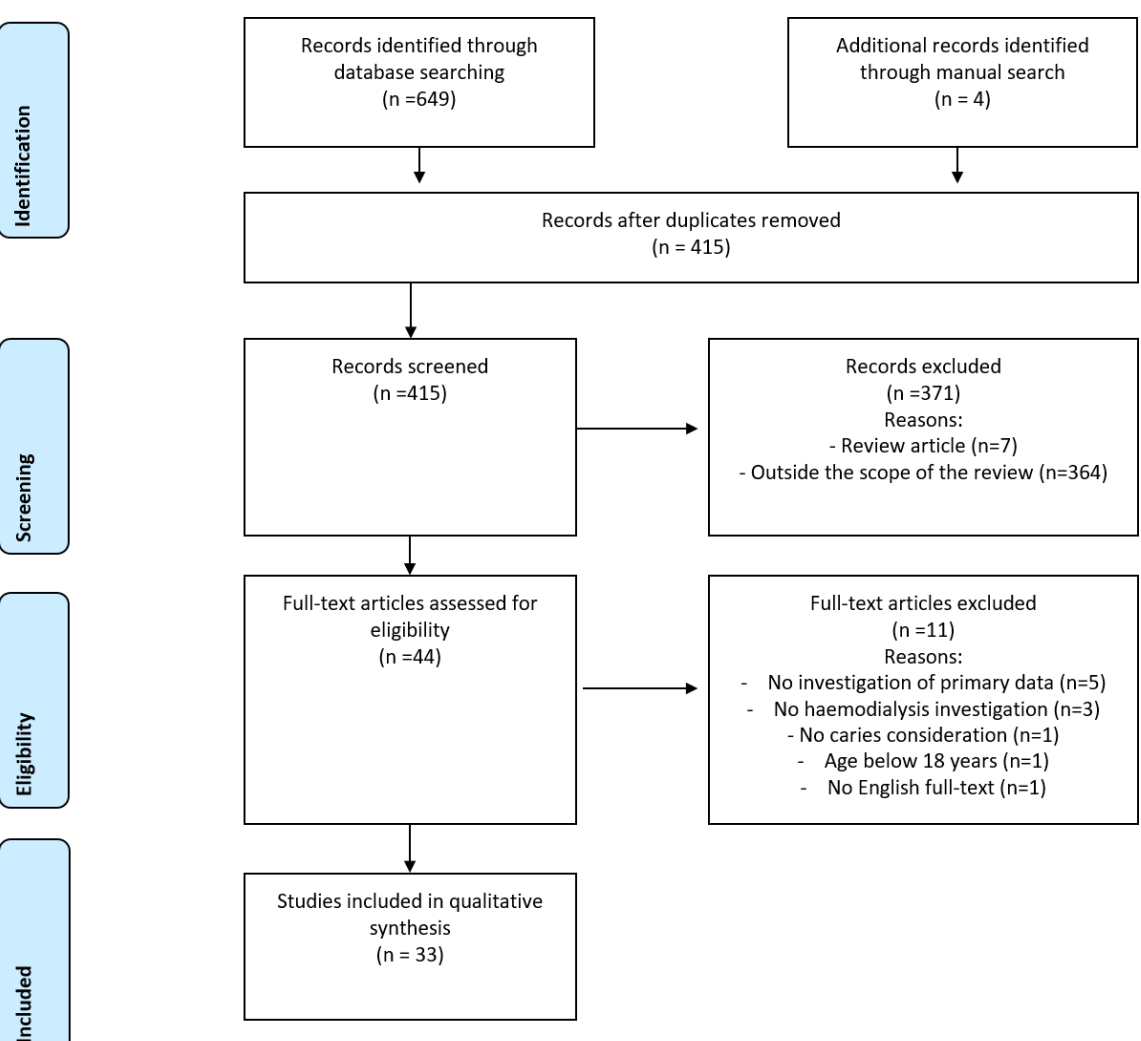
PRISMA diagram for systematic review process.

The search findings according to the PRISMA statement [11] are presented in Figure 1. A total of 653 studies were identified by systematic search complemented by hand search. 238 studies were excluded due to duplications and 371 articles were excluded during screening. The reasons for this were, in seven cases, the type of article (systematic review), while 364 studies were outside the scope of the review. A total of 44 full-text articles were examined regarding their eligibility. Eleven studies were excluded, wherein five studies did not investigate primary data and one study included participants with age below 18 years. Furthermore, in three studies patients did not receive RRT, in one study caries was not considered in the investigation and in one study there was no English full-text available. Accordingly, a total of 33 clinical investigations were included in the qualitative analysis (Figure 1).

### 3.2. Characteristics of Included Studies

Studies from fourteen different countries were included. The majority (31 studies) of all included patients had undergone HD treatment, with a sample size between 28 and 512 participants across studies. Two studies additionally examined patients receiving peritoneal dialysis. The group size was 63 and 286, respectively. Five other studies examined KTx recipients. In these studies, sample sizes between 43 and 286 participants were included. Two studies only investigated patients who received KTx. The sample size amounts were 28 and 44, respectively. Eleven studies investigated a healthy group for comparison. The study type, mean age, sex and disease duration of the included studies are presented in Table 1.

### 3.3. Oral Health Record and Findings

**Figure 2:**
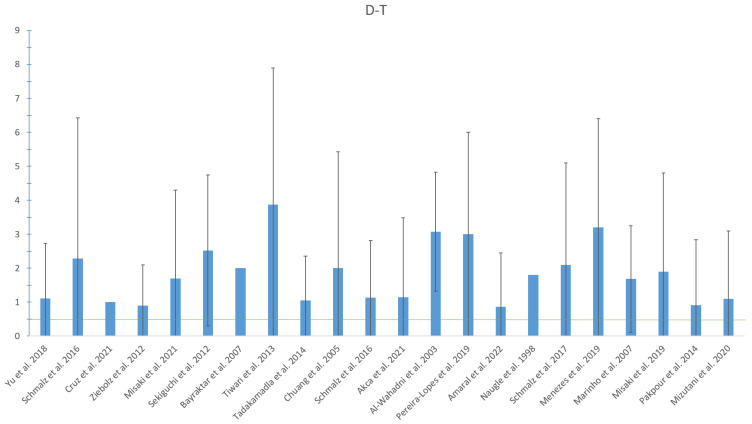
Number of Decayed teeth (D-T) in the respective studies on patients with RRT presented as mean values and standard deviation. The green line reflects the German healthy general population as a national representative value [43] for interpretation of the values.

As shown in Table 2, the applied oral examinations were heterogeneous between the included studies. Dental caries burden was frequently assessed, whereby the decayed-, missing- and filled-teeth index (DMF-T) and its components were examined. All but three studies investigated the DMFT or DT. The number of decayed teeth ranged between 0.7 and 3.87 across studies (Table 2, Figure 2). Several studies examined further oral health parameters, including plaque index (PI) and gingival index (GI), periodontitis severity or community periodontal index (CPI). None of the studies reported on caries activity, stadium/stage or localization (e.g., root caries). Table 2 provides a detailed overview of the oral health parameters examined and, if applicable, the results. Table 3 shows the comparison between RRT patients and healthy individuals in the respective studies. It is obvious that only six out of the 11 studies found significant differences in caries prevalence/incidence between RRT and controls, whereas only four studies confirmed a worse caries burden in RRT individuals (Table 3).

### 3.4. Quality Assessment

The quality appraisal according to AHRQ criteria is presented in Table 4. Most of the included studies were assessed to be of moderate quality (Table 4).

## 4. Discussion

A total of 33 studies from a variety of countries and a large range of sample sizes were qualitatively analyzed in this systematic review. Only one third had a healthy control group, whereby only six revealed a significant difference between RRT and control while four studies showed the RRT group to have higher caries prevalence. To report on caries prevalence, most studies applied the DMF-T index and partly the D-T sub-aspect separately. Number of remaining teeth, salivary as well as RRT-related parameters (e.g., estimated glomerular filtration rate [eGFR], laboratory values) were rarely reported across studies.

Against the previously formed hypothesis, i.e., a higher caries burden in RRT individuals, a strong conclusion cannot be unequivocally formulated. Only four out of the 33 studies found a significantly worse caries prevalence in the RRT group in a direct comparison with a healthy control group, while two studies showed the opposite. However, if the D-T values are listed and compared to available reference values of the healthy general population (Figure 2), a trend can be seen. Within a population-representative sample, the fifth German oral health study (DMSV) showed that adults between 44 and 65 years old (a similar age group as in the included studies) had a D-T of 0.5 [43]. As can be seen from Figure 2, all included studies showed higher D-T values, indicating a worse caries prevalence across studies. However, this interpretation is limited by differences in caries prevalence between different countries [2]. Therefore, the hypothesis is indeed confirmed, but the evidence is still limited. Dental caries was mainly evaluated by DMF-T and D-T across included studies. The DMF-T is an index for clinical studies, whereby the D-T reflects visually detectable carious cavitation on the tooth surface [44]. This is a recommendable clinical examination for research questions, but has several weak points. Early carious lesions without cavitation are not recorded; moreover, caries activity and extent are not assessed in the DMF-T index. Therefore, more comprehensive and detailed procedures which provide such information could be applied, including the International Caries Detection and Assessment System (ICDAS) or the International Caries Classification and Management System (ICCM) [45,46]. Future studies should accordingly consider one of those systems to obtain more detailed information on the caries burden in the patients.

Another issue is the localization of carious lesions, on which the included studies did not provide sufficient information. Especially in individuals undergoing RRT, who might suffer from dry mouth and relevant co-morbidities, the occurrence of root caries would be of certain relevance. Root caries is highly prevalent, especially in individuals with the aforementioned risk factors [47]. Because of the difficult therapy and thus inappropriate prognosis of root carious lesions, its sufficient diagnostic and preventive strategy is eminent [47,48]. Accordingly, the occurrence of root caries in patients on RRT would be of high practical interest; however, none of the included studies evaluated this issue separately, rendering it an important gap in recent evidence. Future studies on dental caries in RRT patients should therefore consider differentiation between different caries stadiums as well as their activity, alongside caries localization, focusing especially on root surfaces. Thereby, additional diagnostic procedures have never been applied to RRT individuals, including, e.g., radiographic diagnostics (bitewing), laser fluorescence or light-induced fluorescence, which have considerable value as diagnostic procedures adjunctive to visual inspection [49,50,51]. Due to differences in sensitivity between visual inspection and adjunctive diagnostics [52], the combination of different diagnostic procedures would be of interest for RRT patients. It would be of particular interest for secondary caries, i.e., carious lesions at the margin of present restorations [53]. Altogether, this discussion underlines the lack of knowledge on dental caries in patients on RRT and provides several novel approaches for future research in the field.

Although the evidence on dental caries in RRT individuals is somewhat unsatisfactory, the increased caries prevalence might be of potential importance for the outcome in those patients. The recent literature suggests some evidence for this relationship. A clinical study on 80 individuals under HD showed that a higher caries burden (DMF-T) was associated with the aortic calcification index and thus with arteriosclerosis (but not with mortality) [54]. Another prospective cohort study found untreated caries to increase all-cause mortality in HD patients [10]. A Taiwanese nationwide population-based cohort study in patients with CKD showed that sufficient root canal treatment, which is often needed in case of fairly advanced carious lesions, leads to a lower risk of death during HD therapy [55]. In the included studies, therapy-related issues (e.g., eGFR) or inflammatory laboratory values (e.g., C-reactive protein [CRP]) were rarely considered across investigations, making a conclusion on this issue impossible. However, the high caries prevalence alongside the potential risk of harm for the patients on RRT supports a demand for improved oral care in those individuals. In the context of the interrelation between oral conditions and kidney failure, several different factors appear of relevance: thereby, those patients are a special cohort with different systemic conspicuities, including increased levels of oxidative stress and inflammation [56], alongside premature ageing of the whole body and vascular system [57,58]. Those issues could be influential for the high oral burden in those individuals, and vice versa. Thereby, patients under RRT show a variety of potential confounding factors for caries development and progression; one relevant issue is the nutrition of patients on RRT, especially considering dialysis-related malnutrition and intake of carbohydrates, which can serve as a substrate for caries-related microorganisms [59,60]. Furthermore, the upper mentioned relevance of reduced salivary flow and quality, especially considering the occurrence of xerostomia, increases the risk of caries, because the reservoir for remineralization is limited [7,61]. In addition, microbiological issues, favoring relevant cariogenic bacteria (especially under consideration of co-morbidities such as diabetes mellitus), are confounding factors in patients on RRT [9]. Finally, patients on RRT are immunocompromised, showing a higher risk of infectious disease in general [62]. The affected immune response could also be a relevant factor for caries development and progression. Alongside gingival recession following periodontal burden, which is highly prevalent in individuals on RRT [63], a remarkable risk for the development of root caries can be presumed. However, the causal relationship or the oral–systemic interrelationship in patients with kidney failure or on RRT still require further research.

Especially under consideration of the patient perspective and therefore within a multidisciplinary, patient-centered approach, this issue has already been addressed previously [11]. This appears to also be relevant for another common oral health issue in RRT individuals which influences caries prevalence: hyposalivation and xerostomia. This issue has rarely been recognized within the studies included in this systematic review. It has been discussed that xerostomia in RRT individuals (especially those on HD) would be important not only for well-being but also for the general morbidity of the patients [64]. Therefore, this deserves a multidisciplinary approach involving nephrologists as well as dentists. Accordingly, a main conclusion of the results from this systematic review is the need for improved, multidisciplinary, patient-centered dental care concepts to improve the dental conditions (and overall oral health) of RRT patients.

This systematic review followed the PRISMA guidelines and had a clear PICO question. The whole systematic search was performed by independent reviewers; furthermore, the research question is of high clinical relevance. However, several limitations need to be addressed for the review itself and the included studies. On the one hand, a variety of heterogeneous studies was included, which were performed in different patients and countries, under application of different, quite general visual caries assessments. This limits the generalizability of the data and is the reason for not performing a quantitative (meta-) analysis, making this current study only a qualitative synthesis. Additionally, studies both with and without a healthy control group were included; the question of whether RRT individuals suffer from higher caries prevalence can only be unequivocally answered in case of a control group. However, many studies addressed the topic without a control group and showed high caries prevalence when compared to available reference values for interpretation (see Figure 2). Furthermore, the included studies were mainly of moderate quality (see quality appraisal). Additionally, many studies investigated patients under HD, while PD and KTx are still rarely examined. Alongside the lack of differentiated caries diagnostics and consideration of additional RRT-related parameters, the available evidence must be seen as limited, requiring further well-performed clinical studies in the field.

## 5. Conclusions

Within the limitations of the systematic review, patients on RRT show a high prevalence of dental caries. Thereby, no information regarding caries stadium, activity and localization is available yet. As observational data suggest a link between oral health and mortality in patients on RRT, improved, multidisciplinary, patient-centered dental care concepts are required to support dental and overall oral health in individuals on RRT, along with a need for further research in the field.

## Figures and Tables

**Table 1:** Studies included in systematic review. Values for age and disease duration are presented as mean value ± standard deviation or mean value (range).

Author, Year	Form of RRT	Country	No. of Patients	Study Type	Subjects Mean Age in Years	Treatment Time			Male (%)	Control Group
						HD	PD	Tx		
Yue et al. 2018 [14]	HD	China	30	monocentric	48.53 ± 12.69	>12 month	n/a	n/a	50	30, 46.50 ± 8.83, 53% men
Schmalz et al. 2016 [15]	HD, Tx	Germany	126 (HD: 87; Tx: 39)	clinical multicentre cross-sectional	HD: 60.98 ± 14.01; KTx: 56.51 ± 11.56	≥5 years	n/a	≥5 years	HD: 62.1; KTx: 48.7	91, 58.31 ± 9.91, 43.1% men
Gautam et al. 2014 [16]	HD	India	206	multicentre cross-sectional	46.79 ± 12.78	69%, <1 year, 13.6%, 1–3 years, 16.5% >3 years	n/a	n/a	81.1	n/a
Cruz et al. 2021 [17]	HD, Tx	Brazil	46	descriptive cross-sectional	18–44: 43.5%; 45–54: 21.7%; 55–80: 34.8%	n/a	n/a	80.4% pre Tx, 19.6% post Tx	71.4	n/a
Ziebolz et al. 2012 [18]	HD	Germany	54	multicentre clinical cross-sectional	63.9 ± 13.0	4.1 ± 3.4 years	n/a	n/a	57	n/a
Misaki et al. 2021 [10]	HD	Japan	80 (13 died during 2 year follow up → 67 surviving)	monocentric	67.3 ± 12.2 (surviving group: 65.8 ± 11.9)	7.6 ± 5.9 years	n/a	n/a	60 (surviving group: 54)	n/a
Sekiguchi et al. 2012 [19]	HD	Brazil	94	monocentric cross sectional	<3 y HD: 56% 20–39 years, 44% 40–79 years; >3 y HD: 36.32% 20–39 years; 63.68% 40–79 years	1.: <36 months; 2.: >37 months	n/a	n/a	<3 years HD: 52; >3 years HD: 56.8	n/a
Cengiz et al. 2009 [8]	HD	Turkey	86	monocentric cross sectional	47.85 ± 14.61	n/a	n/a	n/a	54	41, 44.80 ± 10.22, 52% men
Bayraktar et al. 2007 [20]	HD	Turkey	76	multicentre cross sectional	48 ± 15	17: HD <3 years, 59: HD >3 years	n/a	n/a	47	61, 36% men, 46 ± 18
Tiwari et al. 2013 [21]	HD	India	30	monocentric matched case-control study	35–44: 23.3%; 45–54: 36.6%; 55–64: 40%	n/a	n/a	n/a	93.3	30, 35–44: 23.3%, 45–54: 36.6%, 55–64: 40%, 93.3% men
Tadakamadla et al. 2014 [22]	HD	India	74	monocentric cross sectional	46.27 ± 1.42	n/a	n/a	n/a	64.5	150, 43.14 ± 2.31
Cunha et al. 2007 [23]	HD	Brazil	160	multicentre cross sectional	59 ± 12	average time: 2 years (11 months to 11 years)	n/a	n/a	56.9	n/a
Chuang et al. 2005 [24]	HD	Taiwan	128	monocentric cross sectional	57.5	mean duration 3.72 years (range of 0.1 to 10 years)	n/a	n/a	45.3	n/a
Benderli et al. 2000 [25]	Tx	Turkey	28 (G1: 13, G2: 5, G3: 10)	monocentric cross sectional	18–54	n/a	n/a	G1: 0–6 months after transplantation, 6–12 months after transplantation, >12 months after transplantation	n/a	10
Schmalz et al. 2016 [26]	HD, Tx	Germany	70 (HD: 35; Tx: 35)	monocentric cross-sectional	HD: 56.4 ± 11.1, Tx: 55.8 ± 10.9	5.5 ± 6.4 (<1 year: 13.3%, >1 to 5 years: 38.2%, >5 years: 35.3%)	n/a	>1 to 5 years: 11.4%, <5 years: 88.4%	HD: 60, Tx: 47	n/a
Schmalz et al. 2018 [27]	HD	Germany	190	multicentre cross sectional	64.92 ± 15.7	0–2 years (n = 29), 3–5 years (n = 35), 6–8 years (n = 34), 9–12 years (n = 29), 13–20 years (n = 34), >20 years (n = 29)	n/a	n/a	65	n/a
Akca et al. 2021 [28]	HD	Turkey	150	monocentric cross sectional	58.73 (14.59)	54.67 (47.73) months (at least 6 month)	n/a	n/a	54	n/a
Ruas et al. 2018 [29]	HD	Brazil	567	multicentre cross sectional	49.9 ± 13.7	<5 years: 66%, >5 years 34%	n/a	n/a	58	n/a
Bayraktar et al. 2004 [30]	HD	Turkey	72	monocentric cross sectional	45.05 ± 14.15	32.56 ± 40.17	n/a	n/a	53	50, 43.92 ± 18.80, 48% men
Buhlin et al. 2007 [31]	HD	Sweden	51	monocentric cross sectional	55.3 (13.0)	n/a	n/a	n/a	65	n/a
Al-Wahadni et al. 2003 [32]	HD	Jordan	47	monocentric cross sectional	42.9 ± 12.5	1: HD <1 year, 2: HD 1–3 years, 3: HD >3 year	n/a	n/a	51.06	n/a
Pereira-Lopes et al. 2019 [33]	HD, PD	Portugal	63 (17 HD, 35 PD, 11 PD after HD)	Monocentric cross sectional	HD: 53.8 ± 6.8, PD: 46.6 ± 12.3, PD after HD: 45.3 ± 13.6	33.5 months	PD: 5.8 months, PD after HD: 79.9	n/a	HD: 82.4, PD: 48.6, PD after HD: 45.5	n/a
Bots et al. 2007 [34]	HD, Tx	Netherlands	43 (20 of them Tx during study period	monocentric prospective observation study	men: 54 ± 15.7; women: 48.9 ± 17.2	33 ± 28.6 month at baseline	n/a	13.5 ± 7.1 month before second measurement	69.3	n/a
Amaral et al. 2022 [35]	HD	Brazil	60	monocentric cross sectional	60.23 ± 10.87	41.9 ± 56.57 month (45 pat <48 month, 15 pat >48 month)	n/a	n/a	73.33	n/a
Naugle et al. 1998 [36]	HD	USA	45	multicentre cross sectional	n/a	1. (N = 9) pat haemodialysis <1 y, 2. (N = 22) 1–3 y, 3. (N = 14) >3y	n/a	n/a	n/a	n/a
Schmalz et al. 2017 [27]	HD	Germany	159	multicentre clinical cross-sectional study	Without DM: 66.7 ± 13, with DM: 70.5 ± 10.2	Without DM: 4.4 ± 4.1y; with DM: 3.3 ± 2.7	n/a	n/a	Without DM: 63, with DM: 65	n/a
Souza et al. 2008 [37]	HD, PD, Tx	Chile	286 CKD: 13 (4.5%) predialysis, 158 (55%) haemodialysis, 23 (8.4%) peritoneal dialysis, 92 (32.1%) Tx	monocentric cross sectional	42 ± 13	n/a	n/a	n/a	53	n/a
Menezes et al. 2019 [38]	HD	Brazil	107	monocentric cross-sectional	44.64 (20–87)	36 month	n/a	n/a	61.7	107, 43.97, 55.14%
Marinho et al. 2007 [39]	HD	Portugal	50 pat: 22 pharmacological-dietary treatment pat., 28 haemodialysis pat.	monocentric observational, case-control study	64 ± 11	n/a	n/a	n/a	46	64, 60 ± 11 46.9%
Misaki et al. 2019 [40]	HD	Japan	80	monocentric cross sectional	67.3 ± 12.2	n/a	n/a	n/a	60	76, 66.6 ± 12.1, 57.9% men
Pakpour et al. 2014 [41]	HD	Iran	512	multicentre case-controlled study	57.7 ± 17.01	52.12 ± 29.86 month	n/a	n/a	62.9	255, 55.8 ± 15.9, 62% men
Mizutani et al. 2020 [10]	HD	Japan	207	monocentric prospective observational study with 3 years follow-up	65.9 ± 12.1	64 (33, 115) month	n/a	n/a	65.2	n/a
Rocha et al. 2022 [42]	Tx	Brazil	44	monocentric cross-sectional comparative study	45.07 ± 13.87	n/a	n/a	>6 month	56.82	n/a

n/a: not applicable, HD: hemodialysis, Tx: kidney transplantation, PD: peritoneal dialysis, RRT: renal replacement therapy.

**Table 2:** Oral health, especially cariological and selected further parameters within the included studies.

Author, Year	Tooth Loss, Remaining Teeth, Dentures	Caries	Oral Hygiene Parameters	Glomerular Filtration Rate (mL/min/1.73 m^2^)	Laboratory Parameters		Saliva Parameters (Saliva Flow Rate, pH)		Bacterial-/Metabolism
					CRP (mg/L)	Serum Creatinine (µmol/L)	Saliva Flow Rate	pH	
Yue et al. 2018 [14]	>15	DMFT: 4.36 ± 3.92; DT: 1.11 ± 1.62	PI: 2.13 ± 0.45	<15	3.09 ± 5.15	1041.76 ± 216.93	n/a	8.21 ± 0.44	Proteobacteria, Firmicutes, Bacteroidetes, Fusobacteria, Actinobacteria; heterogeneity of supragingival plaque in CKD patients was higher than in control group
Schmalz et al. 2016 [15]	>6	HD: DMFT: 20.43 ± 5.85, DT: 2.29 ± 4.13; KTx: 17.41 ± 5.51, 0.74 ± 1.43	n/a	n/a	n/a	n/a	n/a	n/a	n/a
Gautam et al. 2014 [16]	n/a	Prevalence: 65.3%	CPI (bleeding 0%, calculus 13.1%, pocket 4–5 mm 44.2%, pocket > 6 mm 39.32)	<15	n/a	n/a	n/a	n/a	n/a
Cruz et al. 2021 [17]	n/a	DMFT median: 20.0, DT median: 1.0	n/a	n/a	n/a	n/a	n/a	n/a	n/a
Ziebolz et al. 2012 [18]	12 patients (22%) of 54 were toothless	DMFT (n = 54) 22.1 ± 6.5, DT (n = 54) 0.7 ± 1.2; DMFT (n = 42) 20.4 ± 6.4, DT (n = 42) 0.9 ± 1.2	PDI: median 1	n/a	n/a	n/a	n/a	n/a	n/a
Misaki et al. 2021 [10]	n/a	DMFT: 18.5 ± 6.7, DT: 1.7 ± 2.6	PI and GI; median PI HD: 2.00, PI C: 1.00; GI HD: 0.29, GI C: 0.19	n/a	0.4 ± 1.6	n/a	n/a	n/a	n/a
Sekiguchi et al. 2012 [19]	>12	DMFT group L: 12.14 ± 5.36, group M: 14.34 ± 4.80; DT group L: 2.52 ± 2.22, group M: 4.68 ± 2.60	PI group L: 1.22 ± 0.56, GI group L: 0.88 ± 0.42; PI group M: 1.17 ± 0.55, GI: 1.00 ± 0.41	n/a	n/a	n/a	n/a	n/a	n/a
Cengiz et al. 2009 [8]	n/a	DMF-T HD: 12.7 ± 8.1; C: 11.7 ± 5.5	PI and GI; PI HD: 2.1 ± 8.1; C: 1.7 ± 5.5; GI HD: 1.9 ± 0.3, C: 1.1 ± 0.2	n/a	n/a	n/a	n/a	n/a	n/a
Bayraktar et al. 2007 [20]	n/a	DMFT median HD: 12.0, C: 15.00, DT median HD: 2.00, C: 1.00	PI and GI; median PI HD: 2.00, PI C: 1.00; GI HD: 0.29, GI C: 0.19	n/a	n/a	n/a	n/a	n/a	n/a
Tiwari et al. 2013 [21]	n/a	DMFT HD: 6.37 ± 4.26, C: 2.35 ± 1.28; DT HD: 3.87 ± 4.02, C: 1.63 ± 0.36	n/a	n/a	n/a	n/a	n/a	n/a	n/a
Tadakamadla et al. 2014 [22]	n/a	DMFT stage2: 1.64 ± 1.70, stage3: 1.51 ± 1.26, stage4: 1.25 ± 1.58, stage5: 1.37 ± 1.46; DT stage2: 1.32 ± 1.25, stage3: 1.13 ± 0.95, stage4: 1.05 ± 1.35, stage5: 1.05 ± 1.31	GI: stage 2: 1.26 ± 0.13, stage 3: 1.76 ± 0.16, stage 4: 2.06 ± 0.43, stage 5: 2.40 ± 0.39, controls: 0.92 ± 0.42	stage1: ≥90, stage2: 60–89, stage3: 30–59, stage4: 15–29, stage5: <15 (or dialysis)	n/a	n/a	n/a	n/a	n/a
Cunha et al. 2007 [23]	n/a	DMFT 26.0 ± 7.7, DT 32.9%	CPI (bleeding/ calculus 29.4%, pocket >4 mm 8.8)	n/a	n/a	n/a	n/a	n/a	n/a
Chuang et al. 2005 [24]	n/a	DMFT diabetics: 19.93 ± 8.19, DMFT non-diabetics: 14.29 ± 9.19; DT diabetics: 2.00 ± 3.42, DT non-diabetics: 1.94 ± 2.48	CPI: non-diabetics: Code 0: 1.2%, Code 1: 1.2%, Code 2: 24.7%, Code 3: 45.9%, Code 4: 23.5% CPI: diabetics: Code 0: 0%, Code 1: 2.3%, Code 2: 20.9%, Code 3: 39.5%, Code 4: 16.3%	n/a	n/a	n/a	n/a	diabetics: 7.97 ± 0.67, non-diabetics: 8.22 ± 0.44	n/a
Benderli et al. 2000 [25]	n/a	Incidence: G1: 1.15, G2: 1.4, G3: 4.3	n/a	n/a	n/a	n/a	n/a	n/a	n/a
Schmalz et al. 2016 [26]	5 of 70 were toothless	DMFT HD: 19.47 ± 5.84, DMFT KT: 17.61 ± 5.81; DT HD: 1.13 ± 1.68, DT KT: 0.58 ± 1.15	PBI: HD: 0.38 ± 0.27, KT: 0.52 ± 0.49	n/a	n/a	n/a	n/a	n/a	yes: 11 different periodontal pathogenic bacteria) most prevalence: Eikanella corrodens and Parvimonas micra > Fusobacterium nucleatum > Tanerella forsythia
Schmalz et al. 2018 [27]	16.90 ± 8.8	DMFT: 20.45 ± 6.8	n/a	n/a	n/a	n/a	n/a	n/a	n/a
Akca et al. 2021 [28]	n/a	DT: 1.15 ± 2.33	gum bleeding: 14.0%	n/a	n/a	predialysis: 8.51 ± 2.75, postdialysis: 3.25 ± 1.48	dryness mouth: 81.3%	n/a	n/a
Ruas et al. 2018 [29]	average number 19.3 ± 8.7	Prevalence: 20.4%	Gingivitis: 20.3%	n/a	n/a	n/a	n/a	n/a	n/a
Bayraktar et al. 2004 [30]	n/a	DMFT: HD: 11.91 ± 8.73, controls 13.22 ± 9.68	n/a	n/a	n/a	n/a	Stimulated saliva HD: 0.69 ± 0.31, controls: 1.64 ± 0.44	Stimulated saliva HD: 8.15 ± 0.72, controls: 7.16 ± 0.75	n/a
Buhlin et al. 2007 [31]	average: 22.9 (7.3)	DMFT: 20.1 ± 6.6	oral plaque index: 46 ± 24%	estimated by creatinine clearance from 24h urine samples	3.58 (0.3–218.0)	727 (247)	n/a	n/a	n/a
Al-Wahadni et al. 2003 [32]	n/a	DMFT: 8.47 ± 2.88, DT: 5.07 ± 1.75	PI: 1.59 ± 0.60; GI: 2.16 ± 0.87	n/a	n/a	n/a	n/a	n/a	n/a
Pereira-Lopes et al. 2019 [33]	n/a	DMFT HD: 12.0 ± 9.0, PD: 13.0 ± 7.0, PD after HD: 15.0 ± 4.0; DT HD: 3.0 ± 3.0, PD: 3.0 ± 3.0, PD after HD: 4.0 ± 3.	VPI (visible plaque index): HD: 85.0 ± 26.0, PD: 69.0 ± 30.0, PD after HD: 60.0 ± 38.0	n/a	n/a	HD: 9.2, PD: 7.0, PD after HD: 9.3	unstimulated HD: 0.3 ± 0.2, PD: 0.4 ± 0.4. PD after HD: 0.4 ± 0.4, stimulated HD: 1.0 ± 0.5, PD: 1.1 ± 1.4, PD after HD: 0.8 ± 0.5	unstimulated HD: 7.3 ±0.7, PD: 7.6 ± 0.6, PD after HD: 7.4 ± 0.5, stimulated HD: 7.8 ± 0.4, PD: 7.8 ± 0.4, PD after HD: 7.8 ± 0.5	n/a
Bots et al. 2007 [34]	n/a	Dia. Treatment: DIAL-base: DMFS 39.1 (26.9), DMFT 13.6 (8.5) DIAL-2yr: DMFS 41.6 (27.8), DMFT 14.4 (8.8); TX: DIAL-base: DMFS 41.9 (26.6), DMFT 14.9 (8.1) TX-2yr DMFS 43.1 (25.3) DMFT 15.5 (7.8)	SOHI	n/a	n/a	n/a	Dia. Treatment: DIAL-base: UWS 0.31 (0.19), SWS 1.18 (0.8) DIAL-2yr: UWS 0.31 (0.18), SWS 1.09 (0.54); TX: DIAL-base: UWS 0.3 (0.21), SWS 1.12 (0.66) TX-2yr UWS 0.44 (0.29) SWS 1.38 (0.84)	Dia. Treatment: DIAL-base: UWS 7.28 (0.52), SWS 7.44 (0.43) DIAL-2yr: UWS 7.1 (0.71), SWS 7.28 (0.57); TX: DIAL-base: UWS 7.36 (0.49), SWS 7.39 (0.42) TX-2yr UWS 6.74 (0.4) SWS 7.0 (0.24)	n/a
Amaral et al. 2022 [35]	n/a	DMFT: 22.55 ± 8.39 (D: 0.86 ± 1.59, M: 18.2 ± 10.99, F: 3.49 ± 4.63)	n/a	n/a	n/a	n/a	n/a	n/a	n/a
Naugle et al. 1998 [36]	n/a	subgroup: 1: 11.88 ± 8.06, 2: 13.57 ± 9.06, 3: 9.87 ± 5.53; grand totals: 11.77 (D: 1.8 M: 4.9 F: 4.8)	SOHI: subgroup: 1: 3.18 ± 1.13, 2: 3.35 ± 1.42, 3: 3.17 ± 1.21; grand totals: 3.241 ± 1.26 (N = 44)	n/a	n/a	n/a	n/a	n/a	n/a
Schmalz et al. 2017 [27]	n/a	DMFT all pat: nDM: 22.3 ± 5.5; DM: 21.9 ± 6.1; DMFT pat with teeth: nDM: 21.2 ± 5.4 (D: 1.4 ± 2.1 M: 12.8 ± 8.6 F: 7 ± 5); DM: 20.4 ± 6 (D: 2.1 ± 3 M: 10.8 ± 7.8 F: 7.7 ± 5.5)	n/a	n/a	n/a	n/a	unstimulated: nDM: 0.23 ± 0.23; DM: 0.16 ± 0.2 stimulated: nDM: 0.5 ± 0.4; DM: 0.42 ± 0.42	unstimulated: nDM: 7 ± 0.9; DM: 6.7 ± 0.7	n/a
Souza et al. 2008 [37]	n/a	DMFT: all pat.: 20.6; Pre 22; HD 21; PD 24; Tx 20	presence of Calculus: 86.7%	n/a	n/a	n/a	n/a	n/a	n/a
Menezes et al. 2019 [38]	n/a	DMFT CKD pat: 14.8 ± 8 (D: 2.9 ± 2.7, M: 11.4 ± 8.7, F: 0.5 ± 1.5); Controls: 16.4 ± 7.7 (D: 3.2 ± 3.2, M: 11.6 ± 8.5, F: 1.6 ± 2.5)	Plaque index: CKD 1.1 ± 0.6; Controls: 1.2 ± 0.8	n/a	n/a	n/a	n/a	n/a	n/a
Marinho et al. 2007 [39]	n/a	DMFT: Pat 17.14 ± 7.79 (D: 1.68 ± 1.57 M: 14.08 ± 9.12 F: 2.34 ± 2.75); controls 15.23 ± 70.7 (D: 2.58 ± 2.48 M: 9.09 ± 7.95 F: 4.32 ± 2.49) DMFT of pat: CRF pat 20.64 ± 6.19 (D:2.36 ± 1.27); TRF pat 14.39 ± 7.91 (D: 1.29 ± 1.62)	simplified Greene and Vermillion oral hygiene index: Pat 10 (26.3%) Grade 0–1, 28 (73.7%) G2–3 (differences between CRF and TRF!), controls 32 (56.1%), 25 (43.9%); Ramfjord calculus index: Pat 19 (50%) G0–1, 19 (50%) G2–3, controls 24 (42.1%), 33 (57.9%)	<60	n/a	n/a	n/a	n/a	n/a
Misaki et al. 2019 [40]	n/a	DMFT: HD pat 19 ± 6.6 (D: 1.9 ± 2.9, M: 8 ± 8.7 F: 9.1 ± 6.5); controls 17.3 ± 6.7 (D: 1.6 ± 2.2 M: 5.2 ± 7.4 F: 10.6 ± 5.5); total number of C4 teeth: HD pat 0.7 ± 1.5; controls 0.2 ± 0.7	n/a	n/a	n/a	n/a	n/a	n/a	n/a
Pakpour et al. 2014 [41]	n/a	DMFT: HD pat 20.06 ± 11.16 (D: 0.91 ± 1.93, M: 11.71 ± 7.68 F: 7.37 ± 8.02); controls 10.57 ± 6.74 (D: 2.51 ± 2.12 M: 6.4 ± 4.21 F: 1.43 ± 1.6)	modified Quigley-Hein index visual plaque index: HD pat 1.92 ± 1.28; controls 1.18 ± 1	n/a	n/a	n/a	n/a	n/a	n/a
Mizutani et al. 2020 [10]	22 (16, 26) mean 19.9 ± 7.1	DT: mean D: 1.1 ± 2.0 F: 8.3 ± 5.3	DI-S: mean 0.99 ± 0.76	n/a	hsCRP 0.16 (0.05, 0.45)	n/a	n/a	n/a	n/a
Rocha et al. 2022 [42]	n/a	DMFT: n/a	n/a	n/a	n/a	n/a	n/a	n/a	n/a

M-T: missing teeth, D-T: decayed teeth, F-T: filled teeth, DMF-T: decayed-, missing- and filled teeth index, PI: plaque index, GBI: Gingiva-bleeding index, GI: gingival index, CPI: community periodontal index, PPD: periodontal probing depth, UWS: unstimulated whole saliva, SWS: stimulated whole saliva, n/a: not applicable, OHI: oral health index.

**Table 3:** Results of studies which compared caries prevalence between patients on RRT and control individuals.

Author, Year	Caries Disease Group	Caries Healthy Control Group	Significant Difference between Disease and Control
Yue et al. 2018 [14]	DMFT: 4.36 ± 3.92; DT: 1.11 ± 1.62	DMFT: 2.28 ± 2.52, DT: 0.10 ± 0.31	yes
Schmalz et al. 2016 [15]	HD: DMFT: 20.43 ± 5.85, DT: 2.29 ± 4.13; KTx: 17.41 ± 5.51, 0.74 ± 1.43	DMFT: 16.76 ± 6.37, DT: 0.01 ± 0.10	yes
Cengiz et al. 2009 [8]	DMFT: 12.7 ± 8.1	DMFT: 11.7 ± 5.5	no
Bayraktar et al. 2007 [20]	DMFT median: 12.0 (9.00-18.00), DT median HD: 2.00 (0.25-3.00)	DMFT median: 15.00 (6.50-21.50), DT median: 1.00 (0.00-3.00)	no
Tiwari et al. 2013 [21]	DMFT HD: 6.37 ± 4.26, DT HD: 3.87 ± 4.02	DMFT: 2.35 ± 1.28, DT: 1.63 ± 0.36	DMFT: yes, DT: no
Tadakamadla et al. 2014 [22]	DMFT: 1.37 ± 1.46; DT: 1.05 ± 1.31	DMFT: 2.24 ± 1.82, DT: 2.19 ± 1.79	DMFT: no, DT: yes
Benderli et al. 2000 [25]	Incidence: G1: 1.15, G2: 1.4, G3: 4.3	Incidence: 1.1	G3: yes
Bayraktar et al. 2004 [30]	DMFT: HD: 11.91 ± 8.73	DMFT: 13.22 ± 9.68	no
Marinho et al. 2007 [39]	DMFT: 17.14 ± 7.79 DT: 1.68 ± 1.57	DMFT: 15.23 ± 7.07, DT: 2.58 ± 2.48	no
Misaki et al. 2019 [40]	DMFT: 19 ± 6.6, DT: 1.9 ± 2.9	DMFT: 17.3 ± 6.7, DT: 1.6 ± 2.2	no
Pakpour et al. 2014 [41]	DMFT: 20.06 ± 11.16, DT: 0.91 ± 1.93	DMFT: 10.57 ± 6.74, DT: 2.51 ± 2.12	yes

**Table 4:** Results of quality appraisal of the included studies, according to AHRQ criteria.

Item	(1) Define the Source of Information (Survey, Record Review)	(2) List Inclusion and Exclusion Criteria for Exposed and Unexposed Subjects (Cases and Controls) Or Refer to Previous Publications	(3) Indicate Time Period Used for Identifying Patients	(4) Indicate Whether or Not Subjects Were Consecutive If Not Population-Based	(5) Indicate If Evaluators of Subjective Components of Study Were Masked to Other Aspects of the Status of the Participants	(6) Describe any Assessments Undertaken for Quality Assurance Purposes (e.g., Test/Retest of Primary Outcome Measurements)	(7) Explain Any Patient Exclusions from Analysis	(8) Describe How Confounding Was Assessed And/or Controlled	(9) If Applicable, Explain How Missing Data Were Handled in the Analysis	(10) Summarize Patient Response Rates and Completeness of Data Collection	(11) Clarify What Follow-Up, If Any, Was Expected and the Percentage of Patients for Which Incomplete Data or Follow-Up Was Obtained	Total Score
Yue et al. 2018 [14]	Yes	yes	no	yes	no	yes	Yes	no	n/a	yes	n/a	6
Schmalz et al. 2016 [15]	Yes	yes	no	yes	no	no	Yes	no	n/a	yes	n/a	5
Gautam et al. 2014 [16]	Yes	yes	yes	yes	no	no	Yes	no	n/a	yes	n/a	6
Cruz et al. 2021 [17]	Yes	yes	yes	yes	no	no	Yes	no	n/a	yes	n/a	6
Ziebolz et al. 2012 [18]	yes	yes	no	yes	no	no	n/a	no	n/a	yes	n/a	4
Misaki et al. 2021 [10]	yes	no	yes	yes	no	no	Yes	yes	n/a	yes	yes	7
Sekiguchi et al. 2012 [19]	yes	yes	no	yes	yes	yes	Yes	no	n/a	yes	n/a	7
Cengiz et al. 2009 [8]	yes	no	yes	yes	no	yes	Yes	no	n/a	yes	n/a	6
Bayraktar et al. 2007 [20]	yes	no	no	yes	no	no	Yes	no	n/a	yes	n/a	4
Tiwari et al. 2013 [21]	yes	yes	no	yes	no	yes	Yes	no	n/a	yes	n/a	6
Tadakamadla et al. 2014 [22]	yes	yes	yes	yes	yes	no	Yes	no	n/a	yes	n/a	7
Cunha et al. 2007 [23]	yes	yes	no	yes	no	yes	Yes	no	n/a	yes	n/a	6
Chuang et al. 2005 [24]	yes	yes	no	yes	no	yes	Yes	no	n/a	yes	n/a	6
Benderli et al. 2000 [25]	yes	no	no	no	no	no	Yes	no	n/a	yes	n/a	3
Schmalz et al. 2016 [26]	yes	yes	yes	yes	no	no	Yes	no	n/a	yes	n/a	6
Schmalz et al. 2018 [27]	yes	yes	no	yes	no	no	Yes	no	n/a	yes	n/a	5
Akca et al. 2021 [28]	yes	yes	yes	yes	no	no	Yes	no	n/a	yes	n/a	6
Ruas et al. 2018 [29]	yes	no	no	yes	no	yes	Yes	no	n/a	yes	n/a	5
Bayraktar et al. 2004 [30]	yes	no	no	yes	no	no	Yes	no	n/a	yes	n/a	4
Buhlin et al. 2007 [31]	yes	no	no	yes	no	no	Yes	no	n/a	yes	n/a	4
Al-Wahadni et al. 2003 [32]	yes	no	no	yes	no	yes	Yes	no	n/a	yes	n/a	5
Pereira-Lopes et al. 2019 [33]	yes	yes	yes	yes	no	yes	Yes	no	n/a	yes	n/a	7
Bots et al. 2007 [34]	yes	no	no	yes	no	no	Yes	no	n/a	yes	n/a	4
Amaral et al. 2022 [35]	yes	yes	yes	yes	no	no	Yes	no	n/a	yes	n/a	6
Naugle et al. 1998 [36]	yes	yes	no	yes	no	yes	n/a	no	n/a	yes	n/a	5
Schmalz et al. 2017 [27]	yes	yes	no	yes	no	yes	Yes	no	n/a	yes	n/a	6
Souza et al. 2008 [37]	yes	no	no	yes	no	no	Yes	no	n/a	yes	n/a	4
Menezes et al. 2019 [38]	yes	yes	no	yes	no	no	Yes	no	n/a	yes	n/a	5
Marinho et al. 2007 [39]	yes	yes	no	yes	no	no	Yes	no	n/a	yes	n/a	5
Misaki et al. 2019 [40]	yes	no	yes	yes	no	no	Yes	no	n/a	yes	n/a	5
Pakpour et al. 2014 [41]	yes	yes	yes	yes	no	yes	Yes	no	n/a	yes	n/a	7
Mizutani et al. 2020 [10]	yes	no	yes	yes	no	no	Yes	no	n/a	yes	yes	6
Rocha et al. 2022 [42]	yes	yes	no	yes	no	yes	Yes	no	n/a	yes	yes	7

## Data Availability

All data generated or analyzed during this study are included in this published article.

## Abbreviations

CKD	Chronic kidney disease
CPI	community periodontal index
CRP	C-reactive protein
D-T	number of decayed teeth
DMF-T	decayed-, missing- and filled teeth index
eGFR	estimated glomerular filtration rate
F-T	number of filled teeth
GI	gingival index
HD	hemodialysis
KTx	kidney transplantation
M-T	number of missing teeth
PI	plaque index
RRT	renal replacement therapy

## References

[1] Kassebaum N.J., Bernabe E., Dahiya M., Bhandari B., Murray C.J.L., Marcenes W. (2015). Global Burden of Untreated Caries: A Systematic Review and Metaregression. J. Dent. Res..

[2] Peres M.A., Macpherson L.M., Weyant R.J., Daly B., Venturelli R., Mathur M.R., Listl S., Celeste R.K., Guarnizo-Herreño C.C., Kearns C. (2019). Oral diseases: A global public health challenge. Lancet.

[3] Wen P., Chen M., Zhong Y., Dong Q., Wong H. (2022). Global Burden and Inequality of Dental Caries, 1990 to 2019. J. Dent. Res..

[4] Pitts N.B., Zero D.T., Marsh P.D., Ekstrand K., Weintraub J.A., Ramos-Gomez F., Tagami J., Twetman S., Tsakos G., Ismail A. (2017). Dental caries. Nat. Rev. Dis. Primers.

[5] Sabharwal A., Stellrecht E., Scannapieco F.A. (2021). Associations between dental caries and systemic diseases: A scoping review. BMC Oral Health.

[6] Cassolato S.F., Turnbull R.S. (2003). Xerostomia: Clinical Aspects and Treatment. Gerodontology.

[7] Bossola M. (2019). Xerostomia in patients on chronic hemodialysis: An update. Semin. Dial..

[8] Cengiz M., Sümer P., Cengiz S., Yavuz U. (2009). The effect of the duration of the dialysis in hemodialysis patients on dental and periodontal findings. Oral Dis..

[9] Rezazadeh F., Bazargani A., Roozbeh-Shahroodi J., Pooladi A., Arasteh P., Zamani K. (2016). Comparison of oral Lactobacillus and Streptococcus mutans between diabetic dialysis patients with non-diabetic dialysis patients and healthy people. J. Ren. Inj. Prev..

[10] Mizutani K., Mikami R., Gohda T., Gotoh H., Aoyama N., Matsuura T., Kido D., Takeda K., Izumi Y., Sasaki Y. (2020). Poor oral hygiene and dental caries predict high mortality rate in hemodialysis: A 3-year cohort study. Sci. Rep..

[11] Schmalz G., Patschan S., Patschan D., Ziebolz D. (2020). Oral health-related quality of life in adult patients with end-stage kidney diseases undergoing renal replacement therapy—A systematic review. BMC Nephrol..

[12] Moher D., Liberati A., Tetzlaff J., Altman D.G., The PRISMA Group (2009). Preferred Reporting Items for Systematic Reviews and Meta-Analyses: The PRISMA Statement. PLoS Med..

[13] Rostom A., Dube C., Cranney A. (2004). Celiac Disease.

[14] Yue Q., Yin F.-T., Zhang Q., Yuan C., Ye M.-Y., Wang X.-L., Li J.-J., Gan Y.-H. (2018). Carious status and supragingival plaque microbiota in hemodialysis patients. PLoS ONE.

[15] Schmalz G., Kollmar O., Vasko R., Müller G., Haak R., Ziebolz D. (2016). Oral health-related quality of life in patients on chronic haemodialysis and after kidney transplantation. Oral Dis..

[16] Gautam N.R., Koganti R., Rao T.H., Agarwal R., Alamanda M. (2014). Effect of end-stage renal disease on oral health in patients undergoing renal dialysis: A cross-sectional study. J. Int. Soc. Prev. Community Dent..

[17] da Cruz A.J.S., de Castilho L.S., Contarini L.C.S., Silva M.E.D.S.E., Abreu M.H.N.G. (2021). Dental Findings of Kidney and Liver Transplantation Patients from a Brazilian Oral Health Care Service. Pesqui. Bras. Em Odontopediatria E Clínica Integr..

[18] Ziebolz D., Fischer P., Hornecker E., Mausberg R.F. (2012). Oral health of hemodialysis patients: A cross-sectional study at two German dialysis centers. Hemodial. Int..

[19] Sekiguchi R.T., Pannuti C.M., Silva H.T., Medina-Pestana J.O., Romito G.A. (2012). Decrease in oral health may be associated with length of time since beginning dialysis. Spec. Care Dent..

[20] Bayraktar G., Kurtulus I., Duraduryan A., Cintan S., Kazancıoğlu R.T., Yildiz A., Bural C., Bozfakioglu S., Besler M., Trablus S. (2007). Dental and periodontal findings in hemodialysis patients. Oral Dis..

[21] Tiwari V., Saxena V., Bhambhal A., Tiwari U., Singh A., Goud S. (2013). The oral health status of patients with renal disease in central india: A preliminary study. J. Ren. Care.

[22] Tadakamadla J., Kumar S., Mamatha G.P. (2014). Comparative evaluation of oral health status of chronic kidney disease (CKD) patients in various stages and healthy controls. Spec. Care Dent..

[23] Cunha F.L., Tagliaferro E.P.S., Pereira A.C., Meneghim M.C., Hebling E. (2007). Oral health of a Brazilian population on renal dialysis. Spec. Care Dent..

[24] Chuang S.-F., Sung J.-M., Kuo S.-C., Huang J.-J., Lee S.-Y. (2005). Oral and dental manifestations in diabetic and nondiabetic uremic patients receiving hemodialysis. Oral Surg. Oral Med. Oral Pathol. Oral Radiol. Endodontology.

[25] Benderli Y., Erdilek D., Koray F., Telci A., Turan N. (2000). The relation between salivary IgA and caries in renal transplant patients. Oral Surg. Oral Med. Oral Pathol. Oral Radiol. Endodontology.

[26] Schmalz G., Kauffels A., Kollmar O., Slotta J.E., Vasko R., Müller G.A., Haak R., Ziebolz D. (2016). Oral behavior, dental, periodontal and microbiological findings in patients undergoing hemodialysis and after kidney transplantation. BMC Oral Health.

[27] Schmalz G., Dietl M., Vasko R., Muller G., Rothermund L., Keller F., Ziebolz D., Rasche F. (2018). Dialysis vintage time has the strongest correlation to psychosocial pattern of oral health-related quality of life—A multicentre cross-sectional study. Med. Oral Patol. Oral Y Cirugía Bucal.

[28] Akça N.K., Arslan D.E., In H. (2021). Examination of factors affecting oral health in patients receiving haemodialysis. J. Ren. Care.

[29] Ruas B.M., Castilho L.S., Carneiro N.C.R., Cardoso N.M.D.M., Reis A.B., e Silva M.E.S., Borges-Oliveira A.C. (2020). Integrality of care for hemodialysis patient in Brazil: An analysis of access to dental care. Ciência Saúde Coletiva.

[30] Bayraktar G., Kazancıoğlu R.T., Bozfakioglu S., Yildiz A., Ark E. (2004). Evaluation of salivary parameters and dental status in adult hemodialysis patients. Clin. Nephrol..

[31] Buhlin K., Bárány P., Heimbürger O., Stenvinkel P., Gustafsson A. (2007). Oral health and pro-inflammatory status in end-stage renal disease patients. Oral Health Prev. Dent..

[32] Al-Wahadni A., Al-Omari M.A. (2003). Dental diseases in a Jordanian population on renal dialysis. Quintessence Int..

[33] Pereira-Lopes O., Simões-Silva L., Araujo R., Correia-Sousa J., Braga A.C., Soares-Silva I., Sampaio-Maia B. (2019). Influence of dialysis therapies on oral health: A pilot study. Quintessence Int..

[34] Bots C.P., Brand H.S., Poorterman J.H.G., Van Amerongen B.M., Valentijn-Benz M., Veerman E.C.I., Ter Wee P.M., Amerongen A.V.N. (2007). Oral and salivary changes in patients with end stage renal disease (ESRD): A two year follow-up study. Br. Dent. J..

[35] Amaral M.A., Miotto A.M.M., Moimaz S.A.S., Garbin C.A.S. (2022). Self-perception of oral health among tertiary-care users: Quanti-qualitative analysis with chronic kidney disease patients. Acta Sci. Health Sci..

[36] Naugle K., Darby M.L., Bauman D.B., Lineberger L.T., Powers R. (1998). The Oral Health Status of Individuals on Renal Dialysis. Ann. Periodontol..

[37] Souza C.M., Braosi A.P.R., Luczyszyn S.M., Casagrande R.W., Pecoits-Filho R., Riella M.C., Ignácio S.A., Trevilatto P.C. (2008). Oral health in Brazilian patients with chronic renal disease. Rev. Med. Chil..

[38] Menezes C., Pereira A., Ribeiro C., Chaves C., Guerra R., Thomaz É.B., Monteiro-Neto V., Alves C. (2019). Is there association between chronic kidney disease and dental caries? A case-controlled study. Med. Oral Patol. Oral Cir. Bucal..

[39] Sobrado Marinho J.S., Tomás Carmona I., Loureiro A., Limeres Posse J., García Caballero L., Diz Dios P. (2007). Oral health status in patients with moderate-severe and terminal renal failure. Med. Oral Patol. Oral Cir. Bucal..

[40] Misaki T., Fukunaga A., Shimizu Y., Ishikawa A., Nakano K. (2019). Possible link between dental diseases and arteriosclerosis in patients on hemodialysis. PLoS ONE.

[41] Pakpour A.H., Kumar S., Fridlund B., Zimmer S. (2015). A case-control study on oral health-related quality of life in kidney disease patients undergoing haemodialysis. Clin. Oral Investig..

[42] Rocha L.C.B., Nunes-Dos-Santos D.L., Costa E.M., Gomes S.V., Rodrigues V.P., Pereira A.L.A. (2021). A Cross-Sectional Study of the Association Between Chronic Oral Disease Burden and Serum Biomarkers in Kidney Transplant Recipients. Prog. Transplant..

[43] Jordan R.A., Micheelis W. (2016). The Fifth German Oral Health Study (DMS V). Institut der Deutschen Zahnärzte (Hrsg.).

[44] WHO (1997). World Health Organization: Oral Health Surveys, Basic Methods.

[45] Ismail A.I., Pitts N.B., Tellez M., Authors of the International Caries Classification and Management System (ICCMS) (2015). The International Caries Classification and Management System (ICCMS™) An Example of a Caries Management Pathway. BMC Oral Health.

[46] Ekstrand K.R., Gimenez T., Ferreira F.R., Mendes F.M., Braga M.M. (2018). The International Caries Detection and Assessment System—ICDAS: A Systematic Review. Caries Res..

[47] AlQranei M.S., Balhaddad A.A., Melo M.A. (2021). The burden of root caries: Updated perspectives and advances on management strategies. Gerodontology.

[48] Meyer-Lueckel H., Machiulskiene V., Giacaman R.A. (2019). How to Intervene in the Root Caries Process? Systematic Review and Meta-Analyses. Caries Res..

[49] Kreher D., Park K.-J., Schmalz G., Schulz-Kornas E., Haak R., Ziebolz D. (2022). Evaluation of quantitative light-induced fluorescence to assess lesion depth in cavitated and non-cavitated root caries lesions—An in vitro study. Photodiagnosis Photodyn. Ther..

[50] Litzenburger F., Schäfer G., Hickel R., Kühnisch J., Heck K. (2021). Comparison of novel and established caries diagnostic methods: A clinical study on occlusal surfaces. BMC Oral Health.

[51] Schwendicke F., Tzschoppe M., Paris S. (2015). Radiographic caries detection: A systematic review and meta-analysis. J. Dent..

[52] Janjic Rankovic M., Kapor S., Khazaei Y., Crispin A., Schüler I., Krause F., Ekstrand K., Michou S., Eggmann F., Lussi A. (2021). Systematic review and meta-analysis of diagnostic studies of proximal surface caries. Clin. Oral Investig..

[53] Brouwer F., Askar H., Paris S., Schwendicke F. (2015). Detecting Secondary Caries Lesions: A Systematic Review and Meta-analysis. J. Dent. Res..

[54] Misaki T., Fukunaga A., Nakano K. (2020). Dental caries status is associated with arteriosclerosis in patients on hemodialysis. Clin. Exp. Nephrol..

[55] Chiu C.-C., Chang Y.-C., Huang R.-Y., Chan J.-S., Chung C.-H., Chien W.-C., Kao Y.-H., Hsiao P.-J. (2021). Investigation of the Impact of Endodontic Therapy on Survival among Dialysis Patients in Taiwan: A Nationwide Population-Based Cohort Study. Int. J. Environ. Res. Public Health.

[56] Ebert T., Neytchev O., Witasp A., Kublickiene K., Stenvinkel P., Shiels P.G. (2021). Inflammation and Oxidative Stress in Chronic Kidney Disease and Dialysis Patients. Antioxidants Redox Signal..

[57] Ebert T., Pawelzik S.-C., Witasp A., Arefin S., Hobson S., Kublickiene K., Shiels P.G., Bäck M., Stenvinkel P. (2020). Inflammation and Premature Ageing in Chronic Kidney Disease. Toxins.

[58] Ebert T., Tran N., Schurgers L., Stenvinkel P., Shiels P.G. (2022). Ageing—Oxidative stress, PTMs and disease. Mol. Asp. Med..

[59] Piccoli G.B., Lippi F., Fois A., Gendrot L., Nielsen L., Vigreux J., Chatrenet A., D’Alessandro C., Cabiddu G., Cupisti A. (2020). Intradialytic Nutrition and Hemodialysis Prescriptions: A Personalized Stepwise Approach. Nutrients.

[60] Ostermann M., Lumlertgul N., Mehta R. (2021). Nutritional assessment and support during continuous renal replacement therapy. Semin. Dial..

[61] Bossola M., Tazza L. (2012). Xerostomia in patients on chronic hemodialysis. Nat. Rev. Nephrol..

[62] Syed-Ahmed M., Narayanan M. (2019). Immune Dysfunction and Risk of Infection in Chronic Kidney Disease. Adv. Chronic Kidney Dis..

[63] Schmalz G., Schiffers N., Schwabe S., Vasko R., Müller G.A., Haak R., Mausberg R.F., Ziebolz D. (2017). Dental and periodontal health, and microbiological and salivary conditions in patients with or without diabetes undergoing haemodialysis. Int. Dent. J..

[64] Wilczynska-Borawska M., Baginska J., Borawski J. (2012). Is xerostomia a risk factor for cardiovascular morbidity and mortality in maintenance hemodialysis patients?. Med. Hypotheses.

